# Prognostic Effect of TTF-1 Expression and Histopathology in the Patients with Advanced Lung Adenocarcinoma Treated with Immunochemotherapy

**DOI:** 10.24546/0100504444

**Published:** 2026-06-02

**Authors:** NATSUHIKO IWAMOTO, JUN YAMADA, NAOE JIMBO, NANAMI YAMASAKI, YUKIHISA HATAKEYAMA, TATSUNORI KIRIU, KANOKO MATSUMURA, MASAHIRO KATSURADA, KEIKO OKUNO, KYOSUKE NAKATA, MOTOKO TACHIHARA

**Affiliations:** 1 Division of Respiratory Medicine, Kobe University Hospital, Kobe, Japan; 2 Department of Diagnostic Pathology, Kobe University Hospital, Kobe, Japan; 3 Division of Respiratory Medicine, Akashi Medical Center, Hyogo, Japan; 4 Division of Respiratory Medicine, Hyogo Prefectural Awaji Medical Center, Hyogo, Japan; 5 Division of Respiratory Medicine, Takatsuki General Hospital, Osaka, Japan; 6 Division of Respiratory Medicine, Hyogo Prefectural Tamba Medical Center, Hyogo, Japan; 7 Division of Respiratory Medicine, Konan Medical Center, Kobe, Japan

**Keywords:** TTF-1, IASLC, Advanced lung adenocarcinoma, Immunochemotherapy

## Abstract

**OBJECTIVES:**

The International Association for the Study of Lung Cancer (IASLC) proposed a grading system based on predominant histologic subtypes and reflecting prognosis. We previously reported that thyroid transcription factor-1 (TTF-1) expression levels and tumor cell proportion are useful predictors of immunochemotherapy for lung adenocarcinoma. Building upon our previous findings that TTF-1 negativity was associated with poorer outcomes in patients receiving immunochemotherapy, this study further investigated the relationship between TTF-1 expression and IASLC histologic subtypes, and analyzed progression-free survival (PFS) according to TTF-1 expression within each histologic subtype.

**MATERIALS AND METHODS:**

We used the same multicenter retrospective dataset as in our previous study, which was conducted between January 2019 and May 2023. TTF-1 was considered positive as a predictive factor of immunochemotherapy when staining showed high TTF-1 expression. The association between TTF-1 expression, histologic subtype, and mucus-producing components was evaluated.

**RESULTS:**

Among 95 patients, the positivity rate for TTF-1 was 61.1%. The negative rate of TTF-1 in mucus-producing adenocarcinomas was significantly higher than that in non-mucus-producing adenocarcinomas (18/29 [62.1%] vs. 18/63 [28.6%]; p < 0.01). As per the analysis of IASLC grade 3, the median PFS of TTF-1 negative patients was significantly worse than that of positive patients (6.0 vs. 7.6 months, p = 0.035). Among patients with solid predominant patterns, the median PFS was significantly worse in the patients with TTF-1 negative than positive (5.2 vs. 8.5 months, p = 0.014).

**CONCLUSION:**

This study shows that the combination of histopathology and TTF-1 expression is a predictive factor in patients treated with combined immunochemotherapy.

## INTRODUCTION

The treatment strategies for advanced or recurrent non-small cell lung cancer (NSCLC) differ from those for adenocarcinoma and squamous cell carcinoma. The current World Health Organization (WHO) classification of lung carcinoma is primarily based on hematoxylin and eosin (H&E) staining. The use of a panel of immunohistochemistry (IHC) stains, including thyroid transcription factor-1 (TTF-1), allows for correct subclassification. When morphological evaluation is inconclusive, tumors with IHC findings characteristic of adenocarcinoma are referred to as NSCLC favor adenocarcinoma. A previous study reported the TTF-1 positivity rate to be 72% (1).

The association between TTF-1 and survival in patients with lung cancer has been investigated in previous studies (2, 3), most of which reported a poor prognostic role of negative TTF-1 expression in lung adenocarcinoma. Currently, combined immunochemotherapy has been developed as the standard treatment for patients with advanced NSCLC (4, 5). Nevertheless, the predictive factors for combined immunochemotherapy in patients with NSCLC are yet to be fully investigated. We have previously reported that the absence of strong and extensive TTF-1 staining was significantly associated with worse outcomes in patients with advanced or recurrent lung adenocarcinoma or NSCLC favor adenocarcinoma treated with pemetrexed-based immunochemotherapy (6).

The International Association for the Study of Lung Cancer (IASLC) recently proposed a new grading system for lung adenocarcinoma (7). The model is a 3-tier grading system based on the predominant histologic pattern combined with high-grade patterns. The system consists of the following categories: Grade 1 (well differentiated), defined by a lepidic predominant pattern; Grade 2 (moderately differentiated), defined by acinar or papillary predominant patterns with no or less than 20% high-grade patterns; and Grade 3 (poorly differentiated), defined as any tumor with 20% or more high-grade patterns (solid, micropapillary, or complex glandular patterns). This grading system has not been validated in variants of adenocarcinoma, including invasive mucinous adenocarcinoma (7). Whether variations in TTF-1 staining in different histologic subtypes are related to prognosis, especially in patients treated with combined immunochemotherapy remains unclear. Treatment strategies for patients with poor prognoses remain an unmet need. Therefore, we investigated any potential association between TTF-1 expression and histopathology based on the efficacy of immunochemotherapy.

## MATERIALS AND METHODS

### Study design and patients

We used the same datasets as in our previous study, which was a multicenter retrospective observational study conducted between January 2019 and May 2023 (6). In the present analysis, we focused on the classification of IASLC histologic subtypes and evaluated the prognoses of patients treated with immunochemotherapy. All patient eligibility criteria, data collection methods, and ethical approvals were as previously described (6). Informed consent was obtained through an opt-out on the website. The study was approved by the Kobe University Ethics Committee (B210309, March 23, 2022) and the committees of all participating institutions. It was conducted in accordance with the Declaration of Helsinki and was registered with the University Medical Hospital Information Network of Japan (UMIN-CTR, registration no. UMIN000046901).

### Outcome

In this study, we further investigated the relationship between TTF-1 expression and IASLC histologic subtypes, and analyzed PFS according to TTF-1 expression within each histologic subtype.

### Tissue samples

We previously collected all unstained slides and tissue samples from each institution and stained them with H&E and TTF-1. The tissues were subtyped on the basis of H&E morphology according to the WHO criteria by an experienced pathologist (NJ). IHC was performed using the same protocol and dataset as described in our previous study (6), which established the prognostic relevance of TTF-1 scoring. Each slide was stained with an anti-TTF-1 antibody (clone 8G7G3/1; 1:100 dilution). Staining intensity and percentage of positive cells were scored as previously described (6). Staining intensity was scored as follows: negative or 0 (Appendix 1A); weak, 1 (Appendix 1B); strong, 2 (Appendix 1C). The percentage of positive cells was also scored (0%, 0; 1–9%, 1; 10–49%, 2; ≥50%, 3). They were classified by combining staining intensity and tumor percentage scores (Appendix 1D). A TTF-1 score of 5 was considered positive, and a TTF-1 score of 4 or less was considered negative. When H&E staining was inconclusive, a TTF-1 score more than 2 was considered NSCLC favor adenocarcinoma.

Adenocarcinoma histologic subtype was defined based on the most predominant pattern according to IASLC, American Thoracic Society (ATS), and European Respiratory Society (ERS) (8). We subdivided tumors into lepidic predominant, acinar predominant, papillary predominant, micropapillary predominant, complex glandular predominant, and solid predominant subtypes. In cases where multiple histologic subtypes coexisted within a single resected specimen, TTF-1 expression was evaluated using the predominant subtype.

H&E staining was evaluated based on the proportion of intracytoplasmic mucin within tumor cells (0%, 1–49%, or ≥50%). Tumors were classified as mucus-producing adenocarcinomas if the mucus-producing component comprised ≥1%.

### Statistical analysis

All statistical analyses were performed using EZR software, version 1.51 (Saitama Medical Center, Jichi Medical University, Saitama, Japan) (9). The relationship among TTF-1 expression, IASLC, and predominant histologic patterns was evaluated by Fisher’s exact test. PFS was calculated using the Kaplan-Meier analysis, and differences were compared using the log-rank test. PFS is described as the median and estimated 95% confidence interval (CI). P values less than 0.05 were considered statistically significant.

## RESULTS

### Patient’s characteristics and prognosis by TTF-1 expression

Ninety-five patients were included in the final analysis: 14 with resected specimens (including 3 from extrapulmonary sites), 74 patients with the biopsy specimens (including 8 from extrapulmonary sites), and 7 with pleural fluid specimens. We had previously reported that the positivity rate of TTF-1 was 61.1%, when a TTF-1 score of 5 was considered to be positive (Appendix 1D). There were no significant differences in patient characteristics between positive and negative TTF-1 groups, excluding the PD-L1 tumor proportion score. The PFS of the TTF-1 positive patients without an oncogene driver mutation was 7.9 months (95% CI: 5.9–12.0), whereas that of the TTF-1 negative patients was 5.9 months (95% CI: 4.8–7.6) (p = 0.04) (6).

### Association between TTF-1 expression and histologic subtypes

**Fig. 1:**
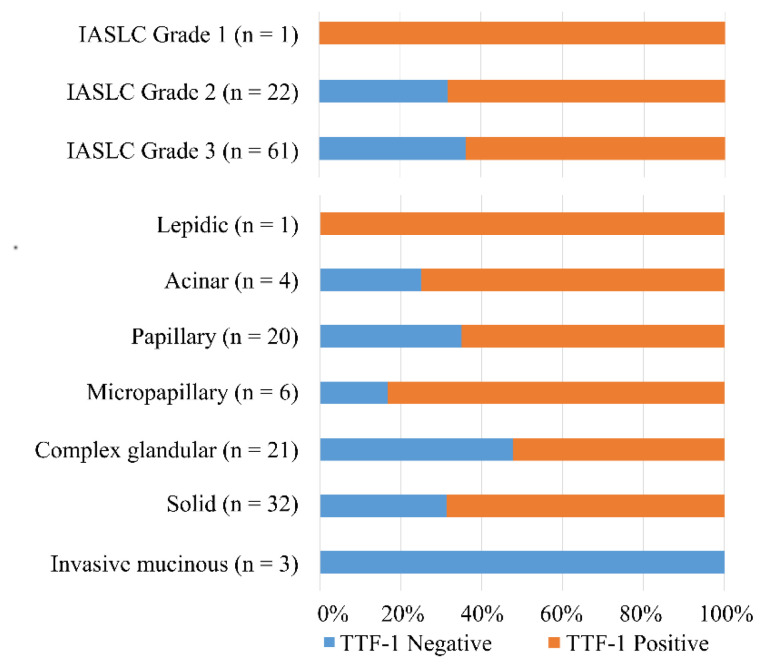
The association between TTF-1 and pathological features. TTF-1 negativity rate for each IASLC grade and for each histologic predominant tumor subtype. IASLC, International Association for the Study of Lung Cancer; TTF-1, thyroid Transcription factor-1.

Evaluation of the predominant histologic pattern in the 21 cases of NSCLC favor adenocarcinoma showed a solid pattern in 13 cases and a complex glandular pattern in 3 cases, while 5 cases were not assessable. The IASLC grades and histopathological characteristics for TTF-1 expression are shown in Table I. IASLC grade 3 was identified in 61 (64.2%) samples. There was no significant association between IASLC grade and TTF-1 expression (Table I, Fig. 1). Among the predominant lung adenocarcinoma subtypes, the solid pattern was the most frequent (32, 33.7%), followed by the complex glandular (21, 22.1%) and papillary (20, 21%) patterns. Patients with complex glandular, papillary, solid predominant patterns tended to have a higher TTF-1 negativity rate (10/21 [47.6%], 7/20 [35%], 10/32 [31.3%], respectively). Three patients with an invasive mucinous predominant pattern were TTF-1 negative. The negative rate of TTF-1 in mucus-producing adenocarcinomas was significantly higher than that in non-mucus-producing adenocarcinomas (18/29 [62.1%] vs. 18/63 [28.6%]; p < 0.01).

**Fig. 2:**
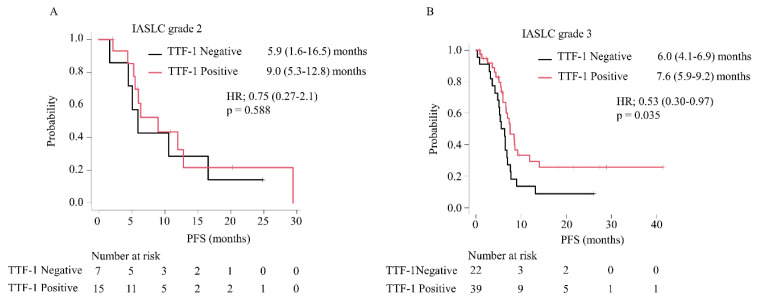
Kaplan–Meier curves of PFS with 95% CI and HR with 95% CI in patients with IASLC grade 2 with TTF-1 positive and negative (A). Kaplan–Meier curves of PFS with 95% CI and HR with 95% CI in patients with IASLC grade 3 with TTF-1 positive and negative (B). IASLC, International Association for the Study of Lung Cancer; TTF-1, thyroid Transcription factor-1; PFS, progression-free survival; HR, Hazard Ratio.

**Fig. 3:**
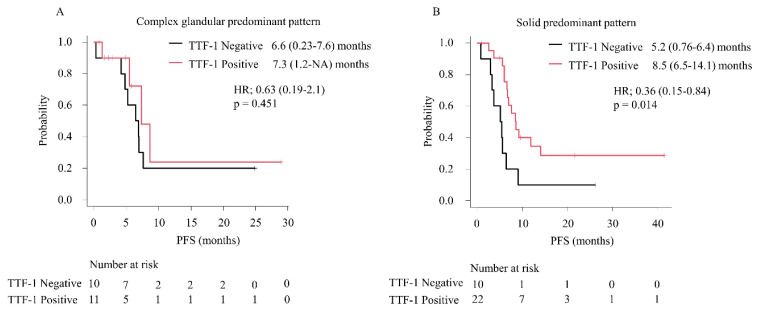
Kaplan–Meier curves of PFS with 95% CI and HR with 95% CI in patients with complex glandular solid predominant pattern with TTF-1 positive and negative (A). Kaplan–Meier curves of PFS with 95% CI and HR with 95% CI in patients with solid predominant pattern with TTF-1 positive and negative (B). IASLC, International Association for the Study of Lung Cancer; TTF-1, thyroid Transcription factor-1; PFS, progression-free survival; NA, Not Applicable; HR, Hazard Ratio.

The median PFS with TTF-1 positivity or negativity following subgroup analysis of IASLC grade and histologic subtypes is shown in Table II. Irrespective of whether TTF-1 expression was negative or positive, in a subgroup analysis of IASLC grade 2, there were no significant differences for the median PFS (5.9 [95% CI: 1.6–16.5] vs. 9.0 months [95% CI: 5.3–12.8], p = 0.588; Table II, Fig. 2A). In contrast, in a subgroup analysis of IASLC grade 3, the median PFS was significantly worse in patients having TTF-1 negative than in those with TTF-1 positive (6.0 [95% CI: 4.1–6.9] vs. 7.6 months [95% CI: 6.0–9.2], p = 0.035; Table II, Fig. 2B). Among patients with a non-solid-predominant pattern, there were no significant differences in the median PFS, regardless of TTF-1 expression. For example, among the patients with complex glandular predominant pattern, there were no significant differences for the median PFS regardless of whether TTF-1 expression was negative or positive (6.6 [95% CI: 0.23–7.6] vs. 7.3 months [95% CI: 1.2–NA], p = 0.451; Table II, Fig. 3A). However, among the patients with solid predominant pattern, the median PFS was significantly worse in those with TTF-1 negative (5.2 [95% CI: 0.76–6.4] vs. 8.5 months [95% CI: 6.5–14.1], p = 0.014; Table II, Fig. 3B). The median PFS in patients with an invasive mucinous pattern was only 2.7 months, although the individual median PFS in patients with other patterns was longer. All three cases with an invasive mucinous pattern were negative for driver mutations, including KRAS.

## DISCUSSION

Our previous study reported the poor prognostic role of negative TTF-1 expression in lung adenocarcinoma in patients treated with combined immunochemotherapy (6). The present study indicates that the patients with IASLC grade 3 had a significantly shorter PFS for TTF-1 negative than that for TTF-1 positive. In contrast, the patients with IASLC grade 2 had PFS for TTF-1 negative comparable to that for TTF-1 positive. These trends were consistent with the analysis to solid or non-solid predominant patterns. These analyses indicate that the prognosis among TTF-1 negative patients with lung adenocarcinoma receiving immunochemotherapy may differ based on the pathological subtypes.

We evaluated the morphological patterns of TTF-1 positive and negative cases. TTF-1 expression is generally negative in poorly differentiated patterns associated with advanced disease (10). Studies in mouse models have revealed that downregulation of TTF-1 would be associated with loss of differentiation, enhanced tumor seeding ability, and increased metastatic proclivity in lung adenocarcinoma (11, 12). This implies that poorly differentiated types may tend to have negative TTF-1 expression in lung adenocarcinoma. An earlier study reported that TTF-1 negativity was an independent predictor of disease recurrence in postoperative patients after adjusting for the IASLC/ATS/ERS classification (13). Although the association between TTF-1 expression and the prognosis of immunochemotherapy has been previously investigated (3), including in our earlier report (6), this is the first study to investigate the association between pathological subtypes in relation to TTF-1 expression and prognosis in patients treated with immunochemotherapy. Therefore, further stratified analysis not only TTF-1 expression but also pathological subtypes analysis would be desirable. The distinction in prognosis of immunochemotherapy based on pathological subtypes in relation to TTF-1 expression is a novel insight of this study.

Previous studies reported that TTF-1 positivity was more likely in tumors with lepidic, minimally invasive, acinar, papillary, and micropapillary predominant patterns, whereas, it was less likely in those with solid, colloid predominant patterns, as well as in invasive mucinous adenocarcinoma (10, 13). It is well accepted that lepidic predominant tumors have the best prognosis, followed by acinar and papillary with intermediate prognosis, and then by solid, micropapillary and complex glandular patterns (14). As per previous reports, the solid predominant pattern was associated with significantly lower TTF-1 expression and involved a more immune-resistant microenvironment than the non-solid predominant pattern (15). The solid predominant pattern has clinicopathologic characteristics with poor differentiation, a higher rate of lymph node metastasis, and a higher pathological stage, accounting for its worse prognosis among patients treated with chemotherapy (15). However, that study did not focus on advanced lung adenocarcinoma under immune checkpoint inhibitor (ICI)-based treatments. We showed that among patients with a solid predominant pattern treated with immunochemotherapy, median PFS was significantly worse in TTF-1-negative cases than in TTF-1-positive ones. Previous reports have shown that the solid predominant pattern is associated with higher PD-L1 expression, increased tumor mutation burden (TMB), and a greater density of CD8-positive tumor-infiltrating lymphocytes (TILs) compared to the non-solid predominant pattern, suggesting that patients with a solid predominant pattern may represent a potential selective group that could benefit from ICI-based treatments (16). Meanwhile, TTF-1 negativity is related to a higher frequency of serine-threonine kinase 11 (STK11) and Kelchlike epichlorohydrin-associated protein 1 (KEAP1) mutations (17, 18). These mutations are known to suppress CD8-positive TILs, and are linked to poor response to ICI-based treatments despite high TMB (19, 20). This may explain the differential response to immunochemotherapy between TTF-1-negative and TTF-1-positive cases within the solid predominant pattern.

In this study, mucus-producing components and TTF-1 expression were mutually exclusive. Previous studies have demonstrated that TTF-1 negativity is associated with mucus-producing adenocarcinoma (21, 22). This finding is consistent with the conclusions of mouse studies conducted by Yutaka et al. (23). It has been reported that TTF-1 negative or weakly positive tumor cells can produce larger amounts of mucus (24). In studies conducted by Zhang et al. (10), all invasive mucinous adenocarcinomas were TTF-1 negative. Similarly, in our study, patients with invasive mucinous patterns were all TTF-1 negative and exhibited poorer prognoses.

Our study had several limitations. First, the sample size was small when the pathological subtypes were classified. IASLC grade and histologic subtypes had to be assessed primarily on biopsy specimens because advanced lung adenocarcinoma is generally not amenable to surgical resection. Although the IASLC grading system was originally proposed for resected specimens, it is considered applicable to small biopsy and cytology samples as well (8). A previous study also utilized biopsy specimens from patients with advanced NSCLC and evaluated IASLC grade and histologic subtypes (25). In our study, some resected specimens exhibited combined histologic subtypes. In such cases, we identified the predominant subtype and assessed TTF-1 expression accordingly. This approach may not fully capture the entire spectrum of histologic components present in the tumor. Moreover, whether the IASLC grading system can be reliably applied to pleural fluid specimens or to NSCLC favor adenocarcinoma remains controversial.

Secondly, this was a retrospective study. Third, the outcomes of immunochemotherapy were not centrally reviewed. Hence, these results need to be validated in future studies.

In conclusion, TTF-1 staining is also important to study the morphology of lung adenocarcinoma cells. The study showed that the combination of histopathology and TTF-1 expression was a predictive factor in patients treated with combined immunochemotherapy.

## Figures and Tables

**Appendix 1:**
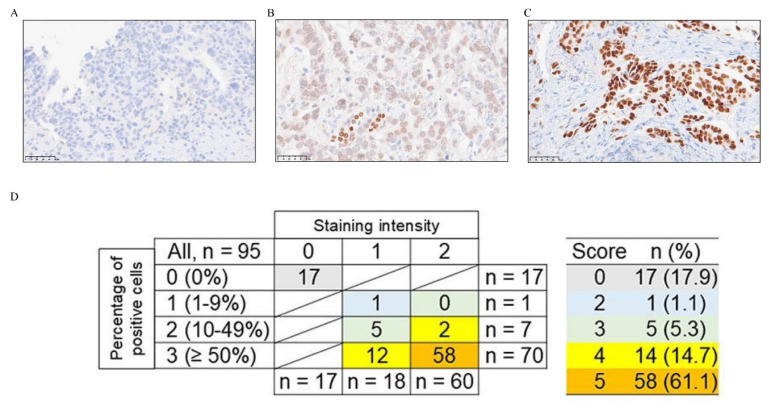
IHC staining of TTF-1. Representatives of TTF-1 staining (clone 8G7G3/1) intensities of (A) negative, (B) weak, and (C) strong. Bar: 50 μm. TTF-1 scoring combined staining intensity and the percentage of stained cells (D). TTF-1, thyroid transcription factor-1; IHC, immunohistochemistry. Source: Ref. 6 (Yamada J, et al.). Reproduced under the Creative Commons Attribution-Non Commercial 4.0 International License (CC BY-NC 4.0).

**Table I:** Histopathology for the expression of TTF-1

	All n = 95 (%)	TTF-1 Positive n = 58 (%)	TTF-1 Negative n = 37 (%)	p value
Tumor Grade (IASLC grade)				
Well differentiated (grade 1)	1 (1.0)	1	0	
Moderately differentiated (grade 2)	22 (23.2)	15 (68.2)	7 (31.8)	
Poorly differentiated (grade 3)	61 (64.2)	39 (64.0)	22 (36.1)	0.868
NA	11 (11.2)			

Histologic subtypes				
Lepidic	1 (1.0)	1 0		
Acinar 4 (4.2)	3 (75)	1 (25)		
Papillary	20 (21)	13 (65)	7 (35)	
Micropapillary	6 (6.3)	5 (83.3)	1 (16.7)	
Complex glandular	21 (22.1)	11 (52.4)	10 (47.6)	
Solid	32 (33.7)	22 (68.8)	10 (31.3)	
Invasive mucinous	3 (3.2)	0	3	0.227
NA	8 (8.4)			

Mucus-producing				
0%	63 (66.3)	45 (71.4)	18 (28.6)	
1–49%	24 (25.2)	9 (37.5)	15 (62.5)	
≥50%	5 (5.2)	2 (40)	3 (60)	<0.01
NA	3 (3.2)			

IASLC, International Association for the Study of Lung Cancer; TTF-1, thyroid Transcription factor-1; NA, Not Applicable.

**Table II:** Efficacy of immunochemotherapy for histopathology and IASLC grade on each TTF-1

	TTF-1 Positive	TTF-1 Negative	HR (95% CI)	p value
		PFS, month (95% CI)		
Tumor Grade (IASLC grade)				
Well differentiated (grade 1)	8.0 (NA–NA)			
Moderately differentiated (grade 2)	9.0 (5.3–12.8)	5.9 (1.6–16.5)	0.75 (0.27–2.1)	0.588
Poorly differentiated (grade 3)	7.6 (5.9–9.2)	6.0 (4.1–6.9)	0.53 (0.30–0.97)	0.035

Histologic subtypes				
Lepidic	8.0 (NA–NA)			
Acinar	5.5 (4.4–NA)	5.0 (NA–NA)	0.41 (0.03–6.6)	0.515
Papillary	12.0 (5.3–NA)	6.4 (1.6–13.1)	0.43 (0.14–1.3)	0.122
Micropapillary	4.4 (0.79–NA)	7.7 (NA–NA)	NA	0.139
Complex glandular	7.3 (1.2–NA)	6.6 (0.23–7.6)	0.63 (0.19–2.1)	0.451
Solid	8.5 (6.5–14.1)	5.2 (0.76–6.4)	0.36 (0.15–0.84)	0.014
Invasive mucinous		2.7 (1.4–NA)		

IASLC, International Association for the Study of Lung Cancer; TTF-1, thyroid Transcription factor-1; NA, Not Applicable.
